# Assessing the relative validity of a web-based self-administered 24-hour dietary recall in a Canadian adolescent’s population

**DOI:** 10.1186/s12937-024-00954-0

**Published:** 2024-06-21

**Authors:** Vicky Drapeau, Catherine Laramée, Jacynthe Lafreniere, Christiane Trottier, Charlotte Brochu, Julie Robitaille, Benoît Lamarche, Simone Lemieux

**Affiliations:** 1 Department of Kinesiology, Faculty of Medicine, Université Laval Québec, 2300, rue de la Terrasse, G1V 0A6 Québec, Canada; 2 School of Nutrition, Laval University, Québec, G1V 0A6 Canada; 3 Centre Nutrition, Santé et Société (NUTRISS), Institut sur la nutrition et les aliments fonctionnels (INAF), Université Laval, G1V 0A6 Québec, Canada; 4 Quebec Heart and Lung Institute Research Center, Université Laval, G1V 4G5 Québec, Canada

**Keywords:** Relative validity, Adolescent, Energy intake, Nutrient intakes, 24-hour dietary recall

## Abstract

**Background:**

Healthy eating habits at a young age are crucial to support growth and development and good general health. In this context, monitoring youth dietary intakes adequately with valid tools is important to develop efficient interventions and identify groups that are more at risk of inadequate intakes. This study aimed to assess the relative validity of the self-administered web-based 24-h dietary recall (R24W) for evaluating energy and nutrient intakes among active adolescents.

**Methods:**

Participants were invited to complete one interviewer-administered 24-h dietary dietary recall and the R24W on up to three occasions within one month. A total of 272 French-speaking active adolescents aged 12 to 17 years from the province of Québec were invited to complete three R24W and one interview-administered 24-h recall. Student’s t-test and correlations were conducted on sex-adjusted data. Percent differences, cross-classification (percentage of agreement), weighted Kappa and Bland-Altman plots were calculated.

**Results:**

Mean (SD) energy intake from the R24W was 8.8% higher than from the interview-administered 24-h dietary recall (2558 kcal ± 1128 vs. 2444 kcal ± 998, *p* < 0.05). Significant differences in mean nutrient intake between the R24W and the interview-administered 24-h dietary recall ranged from 6.5% for % E from fat (*p* < 0.05) to 25.2% for saturated fat (*p* < 0.001), i.e., higher values with R24W. Sex-adjusted correlations were significant for all nutrients except for % E from proteins and thiamin (range: 0.24 to 0.52, *p* < 0.01). Cross-classification demonstrated that 36.6% of the participants were classified in the same fourth with both methods, 39.6% in the adjacent fourth, and 5.7% misclassified. Bland-Atman plots revealed proportional bias between the two methods for 7/25 nutrients. Completing at least two recalls with the R24W increased the precision of intake estimates.

**Conclusion:**

These data suggest that the R24W presents an acceptable relative validity compared to a standard interview-administered 24-h recall for estimating energy and most nutrients in a cohort of French-speaking adolescents from the province of Québec.

**Supplementary Information:**

The online version contains supplementary material available at 10.1186/s12937-024-00954-0.

## Introduction

Developing healthy eating habits at a young age is crucial to support growth and development, as well as good general health [1]. Particularly, adolescence is a critical period where youth experience significant body changes which affect their dietary needs. They are also more independent and make more food choices. Thus, it is important to adequately monitor adolescent dietary intakes to develop efficient interventions and identify groups more at risk of inadequate intakes. In this context, using valid assessment methods is critical to assess dietary intake in this population.

Different dietary assessment methods such as food records, food frequency questionnaires, diet histories, and 24-h dietary recalls have been used among adolescents [2]. However, all these traditional dietary assessment methods rely on trained interviewers for data collection and coding which requires a lot of time and resources for the research team, making them very difficult to apply in large studies.

Researchers have developed new technologies such as web-based tools and smartphone applications, to address this issue. So far, automated self-administered web-based 24-h recalls using a dynamic interface and digital images are among youth’s most widely used dietary assessment tools [3]. For example, the Automated Self-Administrated 24-Hour (ASA24) is a tool developed by the US National Cancer Institute [4] that has demonstrated its validity with youth aged 10 years and older [5]. Vereecken et al. also assessed the validity and acceptability of another computerized 24-h recall, “Youth Adolescent’s Nutrition Assessment on Computer” (YANA-C) [6]. They found that this tool has a good relative validity compared to food records and interviewer-based 24-h dietary recalls. Thus, using web-based automated self-administered 24-h recall seems promising in evaluating dietary intakes in adolescents. Yet, no such tool in French has been validated for this population. Jacques and colleagues (2016) developed a French-Canadian web-based, self-administered 24-h dietary recall (the R24W) that includes multiple passes, during which respondents received cues to help remember and correctly describe foods they consumed the previous day [7]. This tool has already been validated in adults with respect to measured dietary intakes in a laboratory setting [8], biomarkers [9, 10] and reported dietary intakes with a 3-day food record [11] as well as in pregnant women [12]. However, no study has validated this tool among adolescents. Considering the step-by-step approach to recall food intake and its validity in different groups, it is hypothesized that the R24W is also valid among adolescents relative to the interviewer-administered 24-h recall. The present study aimed to assess the relative validity of the R24W for assessing energy and nutrient intakes among French-speaking adolescents using interview-administered 24-h recall as the reference method.

## Methods

### Participants

Adolescents attending a private French high school in Québec City were invited to participate in this study. The school had approximately 750 adolescents aged 12 to 17 years and offered five sports concentration programs embedded within the curriculum: ice hockey, American football, soccer, basketball, or multi-sports. To be included in this study, participants had to be in grades 7, 8 or 9 and be involved in one of the sports concentration programs. Participants also had to have access to the internet at home and not have physical or mental limitations that would limit their ability to recall diet or use a computer.

### Protocol

Participants were part of a larger research project called Winner for Life (*Gagnant pour la vie)* implemented in this high school [13]. This research project aimed to promote positive youth development through a longitudinal life skills training program. This study recruited participants in two waves, one from January to February 2019 and the other from September to October 2019. The present validation study was performed before the intervention phase of the program held in November 2019.

### The self-reported automated web-based recall (R24W)

Participants were invited to complete three recalls with the R24W over a month period. The development of the R24W has been described in detail elsewhere [7]. Briefly, this tool uses a data collection approach inspired by the automated multiple-pass method (AMPM) from the United States Department of Agriculture (USDA) [14]. A total of 2568 food items and 687 recipes (e.g., spaghetti with meat sauce, soup, etc.) are available in the R24W. Food items and recipes are linked to the most recent Canadian Nutrient File (CNF 2015), which allows the automatic extraction of macro- and micronutrient values. Participants had to watch a mandatory tutorial video before using the R24W for the first time. Then, they are guided to recall their previous day’s intakes, meal by meal. After selecting a food item, participants choose the picture that best represents the amount of food eaten. Pictures of up to eight portion sizes are proposed for each food item described by unit and volume. In addition, systematic questions were asked about frequently forgotten food items such as condiments, fats, snacks, and drinks. The R24W also sends automatic emails on randomly chosen dates to notify participants they have a recall to complete. In the first wave of evaluation, participants had to complete the R24W one time in class and two other times at home (using automatic emails). On the second wave of evaluation, participants had to complete the R24W twice in class and once at home to increase the likelihood of having two R24W/participant. The R24W was programmed to be completed on weekdays to ensure a higher completion rate. Except for recalls completed at school, all recall dates were completely random, i.e., participants did not know when they would complete them. The R24W in class was completed at a specific time of day over a 30-min period and supervised by a research team member to answer more technical questions and ensure that all steps were followed correctly. The R24W could be completed at home anytime during the day without assistance and no time limit.

### Interview-administered 24-h dietary recall

On a different day, participants were also invited to perform a short interview to report their 24-h food intake of the previous day. Thirty-nine percent of the participants completed the interview-administered 24-h recall before completing a first recall with the R24W and 61% after completing a first recall with the R24W. The interviews were conducted by registered dietitians (*n* = 4) in a quiet room at school during lunchtime using the USDA Automated Multipass Method (AMPM) [14]. The AMPM uses a five-step, multiple-pass approach to collect dietary information to obtain complete food intake patterns. The first step, the *Quick list*, is a quick and unstructured listing of all foods and beverages consumed the previous day. The second step, *Forgotten Foods*, includes questions probing for commonly forgotten foods in step 1. The third step, *Time and Occasion*, collects the time each food was eaten and the name of the eating occasion. The fourth step is the *Detail Cycle* which includes a description of each food reported, along with quantities and where the food was obtained (if necessary). The final step, the *Final probe*, is a revision of the recall combined with unstructured questions for the other foods recalled and additional memory cues. This method is largely used and has been found to reduce biases in the collection of food intake [15]. Plastic food items and portion sizes (cups, spoons, etc.) were also used to help participants estimate quantities. Each interview lasted between 20 and 30 min. A registered dietitian entered all the interview-administered 24-h recalls into the Nutrific software (Laval University, QC, Canada), linked to the 2015 version of the Canadian Nutrient File database, to obtain the nutritional values. Participants received a gift certificate of $10 at the end of the interview day for this part of the evaluation.

### Other variables

Sociodemographic variables such as age, sex, citizenship, and hours of their concentration sports practiced per week in the school were collected with an electronic sociodemographic questionnaire before completing the R24W or the interview-administered 24-h dietary recall.

### Statistical approach

Overall, 272 adolescents were invited to complete the web-based 24-h dietary recall using the R24W (initial sample). Of these, 111 youth completed at least one web-based 24-h dietary recall and one in-person 24-h recall interview, which were included in the relative validity sample. From the initial sample, the same number completed the web-based 24-hour dietary recall twice, 61 completed the tool at least 3 times and their data were used for the variance analysis. Mean daily intakes and standard deviations for energy and 25 nutrients were assessed with the first R24W completed and the interview-administered 24-h recall. Student’s paired t-test was used to determine whether there was a significant difference between the two methods in assessing each selected nutrient. Pearson correlation coefficient for each nutrient was used to determine the strength of the association between reported intakes using the self-administered R24W and the interview-administered 24-h recall. Student’s t-test and correlations were conducted on sex-adjusted data. Cross-classification (percentage of agreement) and weighted Kappa were calculated to test inter-rater reliability using methods-specific fourths. Bland-Altman plots which show the association between the difference and the mean of two measures were used to evaluate agreement at an individual level across the range of intakes. A significant association demonstrates a proportional bias between these measures [16].

An overview of the relative validity of all nutrients tested was assessed based on criteria proposed by Lombard et al. [17] and also used by Lafrenière et al. for the R24W validation study in adults [11]. Outcome of each test was first categorized as good (G), acceptable (A) or poor (P) and then, agreement between tests and overall relative validity was evaluated by the total of G, A and P validity scores obtained for each nutrient based on the following criteria: G when correlation coefficient ≥ 0.50, cross-classification of ≥ 50% of participants in the same fourth, cross-classification of < 10% of participants in the opposite fourth, weighted Kappa ≥ 0.61, ≤ 10.9% of difference between both methods, non-significant Student’s t-test and slope in the Bland-Altman plot (*P* ≥ 0.05); A when correlation coefficient between 0.20 and 0.49, weighted Kappa between 0.20 and 0.60 and between 11 and 20% of difference between both methods; and P when correlation coefficient < 0.20, cross-classification of < 50% of participants in the same fourth, ≥ 10% of participants in the opposite fourth, weighted Kappa < 0.20, *>* 20% of difference between both methods and Student’s t-test and the slope from the Bland Altman plot were significant (≤ 0.05).

To determine the improvement in precision attributed to multiple 24-h dietary recalls collected with the R24W, we compare the variance from one recall to the average variance of two recalls and the average variance of three recalls in a subsample of 61 adolescents who completed the R24W three times [18]. A ratio *<* 1.00 indicates a lower variance (i.e., greater precision around the mean) and a ratio *>* 1.00 indicates greater variance (i.e., lower precision around the mean) compared with the reference categories (i.e., one day). Log-transformed data were used for not normally distributed data. All statistical analyses were conducted with the software SAS version 9.4 (SAS Institute Inc., NC, USA).

## Results

The study sample of 111 participants comprised more boys (66%) than girls (44%) and had a mean age of 13.2 ± 2,3 years. In addition, participants reported doing an average of 3.6 ± 2.6 h per week of their concentration sport at school. There was no significant difference in the characteristics of participants who completed the R24W for only one day compared to those who completed it for multiple days (data not shown).

### Differences and correlations between the R24W and the interview-administered 24-h recall

Differences in percentage and correlations between one day of self-reported dietary assessment with the R24W and one day of the interview-administered 24-h recall for energy and nutrient intakes are presented in Table 1. The mean values of 21 out of 26 variables assessed with self-reported R24W were within 10% of the mean values obtained with the interview-administered 24-h recall. The largest differences were observed for saturated fat (25.2%) and total sugar (24.0%). Values obtained with the R24W for intake of energy, fat, %E from fat, zinc, saturated fat, and total sugars were significantly different and higher than the corresponding values from the interview-administered 24-h recall (*P* < 0.05). All sex-adjusted correlations were significant (between 0.24 and 0.51, *p* < 0.01) besides %E from proteins and thiamin.

**Table 1:** Average nutrient intakes and correlation coefficients between values derived from the R24W and the interview-administered 24-h recall (*N* = 111)

	R24W		Interview-administered 24-h recall				
	Mean	SD	Mean	SD	% difference^†^		Sex-adjusted correlation (P)
Energy (kcal)	2658.0	1128.0	2444.0	998.1	8.8*		0.52 (< 0.01)
Carbohydrates (g)	347.7	154.7	327.5	142.7	6.2		0.44 (< 0.01)
Fat (g)	100.2	55.6	85.2	40.5	17.6*		0.46 (< 0.01)
Proteins (g)	101.3	39.1	99.5	41.7	1.8		0.46 (< 0.01)
% E Carbohydrates	52.7	9.2	53.9	7.5	-2.2		0.24 (0.01)
% E Fat	32.8	7.8	30.8	7.0	6.5*		0.24 (0.01)
% E Proteins	15.8	4.1	16.5	3.5	-4.2		0.16 (0.09)
Fibres (g)	21.7	12.5	22.6	13.2	-4.0		0.43 (< 0.01)
Vitamin A (mcg)	749.9	583.5	827.1	804.5	-9.3		0.30 (< 0.01)
Thiamin (mg)	2.1	1.0	2.0	1.0	5.0		0.13 (0.16)
Riboflavin (mg)	2.5	1.4	2.3	1.2	8.7		0.49 (< 0.01)
Niacin (mg NE)	46.3	17.8	44.5	17.5	4.0		0.39 (< 0.01)
Vitamin B6 (mg)	1.9	0.8	1.9	0.9	0.0		0.27 (< 0.01)
Folate (mcg DFE)	547.4	288.7	521.0	243.0	5.1		0.27 (< 0.01)
Vitamin B12 (mcg)	5.3	3.3	4.9	3.1	8.2		0.40 (< 0.01)
Vitamin C (mg)	182.9	103.8	207.9	141.9	-12.0		0.43 (< 0.01)
Vitamin D (mcg)	5.7	4.8	5.4	4.2	5.6		0.38 (< 0.01)
Magnesium (mg)	359.9	173.2	346.3	180.2	3.9		0.44 (< 0.01)
Phosphorus (mg)	1695.0	779.2	1600.7	809.5	5.9		0.50 (< 0.01)
Zinc (mg)	13.9	6.3	12.5	6.3	11.2*		0.45 (< 0.01)
Iron (mg)	17.4	8.3	16.9	8.6	3.0		0.51 (< 0.01)
Calcium (mg)	1291.7	770.0	1188.4	738.0	8.7		0.32 (< 0.01)
Potassium (mg)	3241.5	1374.0	3396.1	1670.0	-4.6		0.36 (< 0.01)
Saturated fat (g)	38.3	22.0	30.6	16.0	25.2***		0.43 (< 0.01)
Sodium (mg)	3513.4	1916.0	3748.3	2093.0	-6.3		0.31 (< 0.01)
Total sugars (g)	164.1	77.3	132.3	59.8	24.0***		0.45 (< 0.01)
Mean SD					4.5 8.9		0.38 0.11

DFE, dietary folate equivalent; NE, niacin equivalent ^†^ Calculated as (R24W-interview)/interview*100

* <0.05 ***<0.001

### Cross-classification of the R24W

The cross-classification analysis indicated that, on average, the two methods classified the majority of participants in the same fourth (36.6%) or adjacent fourth (39.6%), with gross misclassification (fourth 1 vs. fourth 4) occurring in 5.7% of participants (Table 2). The mean weighted kappa was 0.26 (Table 2). The Bland-Altman analysis showed a proportional bias for some nutrients (*n* = 7). For fat, %E from carbohydrates, saturated fat and total sugars, differences between the two methods increased with increased mean intakes. In contrast, differences in vitamin A, vitamin C and potassium intakes between the two methods decreased with increased mean intakes (plots in additional file 1).

**Table 2:** Cross-classification of nutrient intakes into fourths of the distribution using either the R24W or the interview-administered 24-h recall (*N* = 111)

	Same fourth (%)	Adjacent fourth (%)	Same or adjacent fourth (%)	Misclassified (1st vs. 4th) (%)	Kappa
Energy (kcal)	46.8	30.6	77.5	3.6	0.36
Carbohydrates (g)	41.4	37.8	79.3	1.8	0.35
Fat (g)	40.5	36.9	77.5	4.5	0.31
Proteins (g)	35.1	47.7	82.9	4.5	0.31
% E Carbohydrates	27.9	40.5	68.5	7.2	0.11
% E Fat	22.5	50.5	73.0	9.0	0.09
% E Proteins	34.2	30.6	64.9	9.0	0.12
Fibers (g)	43.2	36.9	80.2	3.6	0.35
Vitamin A (mcg)	37.8	43.2	81.1	12.6	0.25
Thiamin (mg)	26.1	42.3	68.5	8.1	0.09
Riboflavin (mg)	44.1	38.7	82.9	2.7	0.39
Niacin (mg NE)	36.9	38.7	75.7	4.5	0.26
Vitamin B6 (mg)	34.2	38.7	73.0	7.2	0.19
Folate (mcg DFE)	35.1	37.8	73.0	10.8	0.18
Vitamin B12 (mcg)	37.8	36.9	74.8	2.7	0.28
Vitamin C (mg)	36.9	42.3	79.3	2.7	0.31
Vitamin D (mc)	38.7	37.8	76.6	5.4	0.28
Magnesium (mg)	32.4	49.5	82.0	3.6	0.29
Phosphorus (mg)	43.2	38.7	82.0	3.6	0.37
Zinc (mg)	38.7	39.6	78.4	3.6	0.31
Iron (mg)	37.8	39.6	77.5	7.2	0.26
Calcium (mg)	42.3	34.2	76.6	5.4	0.31
Potassium (mg)	35.1	41.4	76.6	4.5	0.26
Saturated fat (g)	34.2	42.3	76.6	6.3	0.23
Sodium (mg)	34.2	36.0	70.3	9.0	0.16
Total sugars (g)	40.5	39.6	80.2	1.8	0.35
Means	36.6	39.6	76.3	5.7	0.26

DFE, dietary folate equivalent; NE, niacin equivalent

### Relative validity of the R24W

The relative validation assessment using the six tests performed indicates that energy, carbohydrates, proteins, fibres, riboflavin, niacin, vitamin B6, vitamin B12, vitamin C, vitamin D, magnesium, phosphorus, zinc, iron, calcium, potassium, and sodium had results of adequate or good validity (≤ 2 poor outcomes for each nutrient, Table 3). Saturated fat and total sugar were the nutrients with the poorest outcomes (4/7 for both nutrients).

**Table 3:** Statistical test outcomes and proportion of poor outcomes for the relative validity of the R24W (*N* = 111)

	Individual level			Group level			Total of poor outcomes (/7)
	Association	Agreement		Agreement		Presence of bias	
	Correlation coefficient	Cross-classification^†^	Kappa score	% difference	T-test	Bland Altman	
Criteria for good outcome (G)	≥ 0.50	≥ 50%; <10%	≥ 0.61	0-10.9%	*P* > 0.05	*P* > 0.05	
Criteria for acceptable outcome (A)	0.20–0.49		0.20–0.60	11.0–20%			
Criteria for poor outcome (P)	< 0.20	< 50%; ≥10%	< 0.20	> 20%	*P* ≤ 0.05	*P* ≤ 0.05	
Energy (kcal)	G	P-G	A	G	P	G	2
Carbohydrates (g)	A	P-G	A	G	G	G	1
Fat (g)	A	P-G	A	A	P	P	3
Proteins (g)	A	P-G	A	G	G	G	1
% E Carbohydrates	A	P-G	P	G	G	P	3
% E Fat	A	P-G	P	G	P	G	3
% E Proteins	P	P-G	P	G	G	G	3
Fibres (g)	A	P-G	A	G	G	G	1
Vitamin A (mcg)	A	P-P	A	G	G	P	3
Thiamin (mg)	P	P-G	P	G	G	G	3
Riboflavin (mg)	G	P-G	A	G	G	G	1
Niacin (mg EN)	A	P-G	A	G	G	G	1
Vitamin B6 (mg)	A	P-G	P	G	G	G	2
Folate (mcg DFE)	A	P-P	P	G	G	G	3
Vitamin B12 (mcg)	A	P-G	A	G	G	G	1
Vitamin C (mg)	A	P-G	A	A	G	P	2
Vitamin D (mcg)	A	P-G	A	G	G	G	1
Magnesium (mg)	A	P-G	A	G	G	G	1
Phosphorus (mg)	G	P-G	A	G	G	G	1
Zinc (mg)	A	P-G	A	A	P	G	2
Iron (mg)	G	P-G	A	G	G	G	1
Calcium (mg)	A	P-G	A	G	G	P	2
Potassium (mg)	A	P-G	A	G	G	P	2
Saturated fat (g)	A	P-G	A	P	P	P	4
Sodium (mg)	A	P-G	P	G	G	G	2
Total sugars (g)	A	P-G	A	P	P	P	4

DFE, dietary folate equivalent; NE, niacin equivalent; G, Good outcome; A, Acceptable outcome; P, Poor outcome

^†^Cross classification: (G) ≥ 50% in the same fourth; <10% in opposite fourth or (P) < 50% in the same fourth; ≥10% in opposite fourth

### Precision of the R24W

Completing a second and a third self-reported 24-h dietary recalls with the R24W increased the precision of mean intake estimates of all nutrients (Table 4). Adding the second recall to a single measure reduced the mean variance by an average of 36% while adding a third recall further reduced the mean variance by another 9%. Moreover, for some nutrients like riboflavin, folate, and total sugar, adding the third recall reduced the variance by more than 15% compared with the average of only two recalls.

**Table 4:** Variance ratios among combinations of self-reported nutrients intakes assessed using repeated recalls with the R24W.

	Variance ratio † (N=111)†(N=11)		Variance ratio † (N=61)
	2 recalls vs. 1	3 recalls vs. 1	
Energy (kcal)	0.57	0.50	
Carbohydrates (g)	0.68	0.56	
Fat (g)	0.43	0.35	
Proteins (g)	0.64	0.61	
% E Carbohydrates	0.61	0.57	
% E Fat	0.53	0.42	
% E Proteins	0.71	0.60	
Fibres (g)	0.48	0.52	
Vitamin A (mcg)	0.46	0.41	
Thiamin (mg)	0.68	0.55	
Riboflavin (mg)	1.33	0.87	
Niacin (mg NE)	0.69	0.58	
Vitamin B6 (mg)	0.86	0.72	
Folate (mcg DFE)	0.67	0.49	
Vitamin B12 (mcg)	0.53	0.43	
Vitamin C (mg)	0.56	0.55	
Vitamin D (mcg)	0.57	0.49	
Mg (mg)	0.60	0.50	
P (mg)	0.58	0.49	
Zn (mg)	0.57	0.54	
Fe (mg)	0.68	0.71	
Ca (mg)	0.67	0.56	
K (mg)	0.61	0.60	
SFA (g)	0.57	0.51	
Na (mg)	0.59	0.45	
Total sugars (g)	0.86	0.69	
**Mean**	0.64	0.55	
**SD**	0.17	0.11	

DFE, dietary folate equivalent; NE, niacin equivalent ^†^ A ratio *<* 1.00 indicates a lower variance (i.e., greater precision around the mean) and a ratio *>* 1.00 indicates greater variance (i.e., lower precision around the mean) compared with the reference categories (i.e., one recall)

## Discussion

This study assessed the relative validity of a web-based, self-administered 24-h recall (the R24W) relative to an interview-administered 24-h recall among active adolescents aged 12–17 years. Overall results indicate that the R24W has an acceptable level of relative validity for most nutrients and total energy intake. Accordingly, differences in mean nutrient intake between the R24W and the interview-administered 24-h recall were low (< 10%) while correlations between the two methods were significant for most nutrients. Cross-classification demonstrated that more than 75% of the participants were classified in the same or adjacent fourths, and proportional bias between the two methods was observed for only seven nutrients out of 25. Completing a second recall with the R24W significantly reduced the variance by an average of 36%.

In the present study, differences in mean intakes between the R24W and interview-administered 24-h recall were not significant for most nutrients, yet slightly higher energy intake (8%) and consequently higher intakes in fat, saturated fat, zinc, and total sugar intakes were observed with the R24W. Correlations between both methods were significant for most of the nutrients. Considering that digital systems and interview-administered methods typically underestimate caloric and nutrient intakes in children and adolescents [2, 19], the R24W likely underestimates these intakes to a lesser extent than the interview-administered recalls. These observations are similar to other studies aimed at validating web-based dietary assessment tools in adolescents. For instance, Baker et al. (2014) found in a population of young athletes (14–20 years old) that a digital dietary analysis tool for athletes (DATA) had a good relative validity for estimating 24-h energy, carbohydrate, protein, total fat, water and some micronutrient intakes (e.g., sodium, calcium) when compared to an interview-administered 24-h recall using the USDA’s five steps multiple pass method [20]. They also noted that their web-based tool overestimated some nutrients (i.e., total energy, lipid, and calcium intakes) compared to direct dietary observations. Lindroos et al. also reported comparable dietary intake estimates between a web-based dietary recall compared to an interview-administered 24-h dietary recall with adolescents aged 11 to 18 and noted slightly higher energy (+ 210 kcal or 10%) and fat (e.g. +10 g total fat/day) intakes for their web-based dietary recall [21]. Of note, in this last study, the correlations between intakes from the web-based tool and energy expenditure assessed with accelerometers were higher than for the interview-administered recall suggesting lower dietary reporting bias with technologies in adolescents.

The slightly larger discrepancies in energy intake and a few other nutrients (e.g., fat, saturated fat, sugar) observed between the two methods in our study may be attributed to the R24W not being completed on the same day as the in-person 24-hour recall interview. For instance, other research, such as the YANA-C (Youth Adolescents’ Nutrition Assessment on Computer) for adolescents (mean age 14.6 ± 1.7), has shown smaller differences in energy intake (3% lower) and fat intake (5% lower) when a web-based 24-hour recall is conducted on the same day as an in-person interview, demonstrating good validity [22]. Bradley et al. observed that mean intakes for all assessed macronutrients and micronutrients in a younger group (ages 11–16) were within 10% of those reported in interviewer-led recalls, and within 3% for the older group (ages 17–24) [23]. Albar et al. reported very good validity for Myfood24, another web-based 24-hour recall tool, in an adolescent population (ages 11–18) when completed twice on the same day compared to an interviewer-administered recall [24]. Completing the assessment tool on the same day appears to improve the accuracy and reliability of dietary intake measurements. However, this approach can also introduce recall bias. For instance, if participants begin with an interview-administered 24-hour recall, where a nutritionist helps them recall their intake using specific techniques (e.g., reconstructing their day or asking about leftovers from the night before), it could artificially simplify subsequent completion of the R24W, as participants would have had prior “training” to respond accurately. This might not accurately reflect typical, real-life dietary reporting. To mitigate this in our study, the R24W and interviewer-administered recalls were conducted on separate days, reducing the potential for recall bias. This methodology has demonstrated good concordance across most nutrients, indicating that despite the timing differences between assessments in various studies, the consistency observed supports the reliability of web-based dietary recalls in accurately capturing nutrient intakes compared to more traditional methods.

The level of agreement for most of the nutrients between the two dietary recalls in the present study was also high: intake of more than 75% of nutrients was classified into the same or the adjacent fourths, only 5.7% were misclassified and the mean weighted kappa was considered good for most nutrients. Bland-Altman plots revealed only a few biases, particularly for higher intakes, but most data points fell within the limits of agreement. These good levels of agreement align with studies from Lindroos et al. [21] and Albar et al. [24] who observed good levels of agreement using intraclass correlations between web-based assessment tools and interview-administered 24-h recall for similar populations. Moreover, our results are consistent with a validation study of the R24W in adults, where 80% were classified in the same or adjacent fourths and fewer than 10% in opposite fourths, with only a few outliers identified [11]. It is important to note that complete agreement between the R24W and the interview-administered 24-hour recall was not expected, as the data were collected on different days using two self-reported methods. Each method is associated with some measurement error, which can be explained by within-person day-to-day variations between groups.

Overall, the relative validity for most nutrients can be described as good or acceptable when evaluated with the criteria proposed by Lombard et al. [17]. The two nutrients for which relative validity was considered lower were saturated fat and total sugar intakes and the poor validity outcomes were mainly related to criteria of agreement at the group level. Since all self-reported dietary assessment tools have limitations and biases, it is not possible to determine which of the two 24-h recalls deviates the most from true intakes for these specific nutrients. Moreover, although the interview-administered 24-h recall is considered the “gold standard” (less biased tool) compared with FFQs and dietary records, there might also be errors attributable to the interview context where interviewer-administered dietary assessment methods may increase social desirability bias, which is the tendency to underreport “bad” foods, i.e. most often high in fat and sugar [25]. Social attitudes about health-related topics such as which foods are considered healthy or not have been observed at a very young age [26]. The lower saturated fat and total sugar intakes observed with the interview-administered 24-h recall compared to the R24W in the present study could reflect this bias. In support of this hypothesis, the Canadian Community Health Survey found similar results regarding saturated fat and sugar intakes in adolescents of the same age group with an interview-administered 24-h recall (mean total fat 27.5 g/day and sugar 119.5 g/day intakes) [27]. Lower intakes for these two nutrients with the interview-administered 24h recall are also consistent with what has been observed using the same R24W compared to an interview-administered 24-h recall in the adult population [28]. Overall, our results suggest caution when interpreting data on saturated fat and total sugar intakes among adolescents when obtained using 24-h recalls, whether administered by an interviewer or through the Web. Further studies in adolescents using additional validation standards, such as incorporating biomarkers or more frequent cross-references with established dietary assessment tools, are needed to enhance the overall validity of dietary assessments.

The present study highlights the importance of using at least two recalls with the R24W for assessing mean nutrient intakes in active adolescents. Accordingly, adding the second recall explained the greatest gain in precision (36%). Adding a third recall increased precision by 9%. Similarly, Brassard et al. have found that using crude data from three recalls collected with the R24W increased the precision of estimates and modified distribution of intakes in a sample of adults compared with using data from only one recall [18]. Thus, using at least two R24W should be recommended to increase precision and the chance to assess the real impact of an intervention on dietary intakes, including in adolescents. However, the present study also highlights the challenge of completing multiple self-administered web-based recalls in adolescents. To increase the likelihood that participants complete the R24W at least two times, we had to plan a specific period in class to complete the R24W. Considering the challenges of assessing dietary intakes of adolescents even when using a self-report web-based 24-h recall, it is also important to have support from the school staff and direction to implement such assessment tools into nutrition research with adolescents.

### Strengths and limitations

This study was realized in a real-life setting and represents one of the few validation studies using a validation process based on the results of six tests to get an overview of the validity of each nutrient. The results from the present study contribute to demonstrating the validity of the R24W in other populations, i.e., adolescents, along with validation studies in adults and pregnant women [8–12]. However, the adolescents in this study were more active than average and were recruited from a private school, which limits the generalization of these results to the entire Quebec adolescent population. Furthermore, because this study compared two self-reported instruments, it is impossible to assess the extent of underestimation or overestimation with the R24W. Weight and height were not measured in the present study, so comparing participants’ reported energy intake to their estimated energy requirement was not possible. Moreover, some bias could be more specific to the R24W system. For instance, the R24W offered more visual aids for reporting portion size, with up to 8 pictures per food item, compared to the interview-administered 24-hour recall, which uses a limited number of food models. The R24W system’s constraints on recipe selection, unlike the more flexible verbal free-listing method of the interview-administered recalls, might not fully capture the variety and specificity of foods consumed by participants. These limitations could lead to inaccuracies in nutrient intake data; for instance, larger portions or generic recipes might include additional components such as sugars, fat spreads, or sauces that participants did not actually consume, potentially leading to overestimating certain nutrients like fats and sugars. These constraints could indeed explain the differences observed in some nutrient estimates between the two methods. Therefore, it will be important to conduct other validation studies in a larger sample of adolescents across different populations, including variables such as body weight and height to confirm to increase the generalization of our results. A larger sample size could also permit subgroup analyses to explore factors (e.g., age) influencing dietary recall accuracy and reliability across different populations. Lastly, qualitative research, such as participant interviews, could be conducted to better understand the discrepancies between the R24W and interview-administered methods. This could elucidate how interactions with the digital system influence reporting, thereby providing insights into these discrepancies and help to improve the reliability of tools like the R24W.

## Conclusion

This study assessed the relative validity of the web-based, self-administered 24-h recall (R24W) in French-Canadian active adolescents in school- and home-based environments. Using six different statistical tests, results showed that, compared to interview-administered 24-h recall, the R24W has an acceptable level of relative validity for most nutrients and energy. Results also indicated that completing at least two recalls with the R24W should be encouraged to reduce random error and increase precision in estimating energy and nutrient intakes in adolescents. This study supports the validity of a French self-administered web-based recall, which offers many advantages in epidemiologic and intervention studies targeting active adolescents, such as standardization of the questions, cost-effective data collection, easy data processing, flexibility, and increased confidentiality. However, data on saturated fat and total sugar intakes should be interpreted cautiously, considering the lower relative validity obtained for these two nutrients in the present study.

### Electronic supplementary material

Below is the link to the electronic supplementary material.

Supplementary Material 1

## Data Availability

The datasets analysed during the current study are available from the corresponding author at reasonable request and are pending approval from the authors as well as the funding agency.

## Abbreviations

R24W: web: based
self: administered 24-h recall
