# Supplementary material for: Assessing the relative validity of a web-based self-administered 24-hour dietary recall in a Canadian adolescent’s population
Source: Nutr J. 2024 Jun 21;23:66. doi: 10.1186/s12937-024-00954-0 (PMC11191218; doi:10.1186/s12937-024-00954-0)
Supplement: Supplementary file 1 — Supplementary Material 1 [file 12937_2024_954_MOESM1_ESM.docx]

**Additional file 1**

Fig. 1. Bland-Altman plot for the agreement between fat intakes assessed from the interview-administered 24-h dietary recall and the R24W, d=15.0; r=0.33; P<0.05; limits of agreements= -94.5 to 124.5

Fig. 2. Bland-Altman plot for the agreement between percentage of energy from carbohydrate intakes assessed from the interview-administered 24-h dietary recall (I24HDR) and the R24W, d=-1.14; r=0.21; P=0.02; limits of agreements= -21.4 to 19.1

Fig. 3. Bland-Altman plot for the agreement between vitamin A intakes assessed from the interview-administered 24-h dietary recall (I24HDR) and the R24W, d=-77.2; r=-0.32; P<0.05; limits of agreements= -1762.9 to 1608.6

Fig. 4. Bland-Altman plot for the agreement between vitamin C intakes assessed from the interview-administered 24-h dietary recall (I24HDR) and the R24W, d=-25.0, r=-0.34; P<0.05; limits of agreements= -281.7 to 231.7

Fig. 5. Bland-Altman plot for the agreement between potassium intakes assessed from the interview-administered 24-h dietary recall (I24HDR) and the R24W, d=-154.6, r=-0.21; P=0.03; limits of agreements= -3451.4 to 3142.2

Fig. 6. Bland-Altman plot for the agreement between saturated fat intakes assessed from the interview-administered 24-h dietary recall (I24HDR) and the R24W, d=7.7, r=0.34; P<0.05; limits of agreements= -33.7 to 49.1

Fig. 7. Bland-Altman plot for the agreement between total sugar intakes assessed from the interview-administered 24-h dietary recall (I24HDR) and the R24W, d=31.8, r=0.28; P<0.05; limits of agreements= -112.4 to 175.9
